# The Plaster of Paris Jacket in Spinal Diseases

**Published:** 1877-12

**Authors:** 


THE PLASTER OF PARIS JACKET IN SPINAL DISEASES.—The repeated inquiries made by physicians respecting the use of plaster of Paris in constructing supporters for spinal disease, show a widely diffused interest in the subject. I have therefore prepared this article to answer the numerous questions addressed me from different sources.

The therapeutic principles involved in the use of the plaster of Paris jacket, are the same as for other spinal supporters, and may be stated as follows:

1. Pott's disease is an inflammation limited almost invariably to the bodies of the vertebræ, and not affecting the articular processes behind them.

2. Hence it is that the bodies only soften and yield to the compression of the vertebræ above, while the articular processes behind remain firm and unchanged, in consequence of which the spine bends at an angle or a short curve, with its concavity forward and its convexity backward.

3. Curative apparatus acts in three methods, viz: (*a*) as a splint to correct or arrest the deformity; (*b*) as a means of securing immobility; (*c*) as a lifting power to take off the weight resting on the diseased vertebræ.

Any firm, broad splint applied to the back may act favorably to prevent the further flexion, and to secure immobility. The action of the plaster of Paris jacket in this respect, like that of all other supporters, is too obvious to require explanation. There is, however, one curative element in the splint principle which is very generally overlooked. Inflammation of the vertebral bodies is usually kept up solely by mechanical irritation; that is to say, by the jarring, the rocking and the pressure of the vertebræ above pressed down by the entire weight of the superior portion of the body. If this mechanical irritation be taken off, almost all cases will recover from the inflammation spontaneously. Now let it be borne in mind that the articular processes are free from inflammation, and are located on a line considerably posterior to the bodies of the vertebræ.

It is evident at a glance, therefore, that if any supporter is so constructed that by its splint power it shall flex the spine firmly backwards, it will throw the weight of the vertebræ upon the articular processes, and take it off from the inflamed bodies of the affected bones.

The direct lifting power of spinal supporters is often overestimated. In small children it amounts to almost nothing, because their hips are smaller than their waists, and there is no basis on which to rest a support for the lifting power. In adults, however, and especially in women, the expansion of the hips gives a counter support, and lifting becomes possible. It may be well to remark, just here, that the old attempt to lift by pressure in the axillæ is abandoned by all good surgeons. A lifting force applied there with sufficient vigor to accomplish anything, soon paralyzes the arms. In spite of this fact, however, the instrument sellers continue to vend, and physicians to buy, absurd pieces of apparatus, even for young children with no expansion of the hips, pretending to lift by crutch pieces in the axillæ.

The only lifting power possible (except an occasional head-stretch) is by embracing the double cone of the waist in something resembling a corset, and this the plaster of Paris jacket accomplishes in cases of adults. The principle is simply this: The waist being the smallest part of the body, anything like a firm, well fitted corset, or the plaster jacket firmly laced on, tends strongly to crowd the cone of the hips downward, and the inverted cone of the rest of the body upward, thus making a decided extension on the middle of the spinal column. It is for this reason that firm corsets, well laced, are a comfort, and actually a benefit to women with incipient Pott's disease. The plaster of Paris jecket acts both as a corset and as a splint, thus fulfilling both indications, acting in that respect like the combination brace, which I have been for years in the habit of applying to cases of Pott's disease.

The most approved method of applying the plaster jacket, requires the following articles to be prepared:

1. A pair of pulleys to suspend the patient.

2. A suspension bar, or rather a sort of whippletree of steel or strong wood, suspended by the pulleys in the centre, and having two notches or hooks on each side, one five inches and one eight inches from the centre.

3. A sort of band or collar of soft stuffed leather to surround the base of the skull, capping the end of the chin in front and passing beneath the occiput behind. Straps pass up from this on each side to the suspension bar.

4. A pair of axillary pads or bands suspended from the centre notches of the suspension-bar.

5. A quantity of roller bandages of coarse cross-barred crinoline, whose meshes are rubbed full of good, dry, fresh plaster of Paris.

The patient is now slung up by the arm-pits so as to hang from the suspension-bar on the axillary bands. To give the spine itself a special extension, the head-sling is also applied and buckled up so as to sustain part of the weight of the body. The axillary bands should be connected together by cross straps, so that by no accident can the child slip down out of them, leaving the whole weight to come on the neck.

A common knit shirt should be on the patient's body, and the plaster of Paris bandages should have already been placed in warm water to soak. It is also well to place a strip of cotton wadding along the more prominent portion of the spine, to prevent pressure on subcutaneous points of bone.

Now, commencing as low down as can be done without impeding the flexion of the thighs, the bandages should be applied over the whole trunk as high as the axillæ, in several successive layers. Sometimes it is well to enclose in them a number of perpendicular strips of perforated tin to obtain additional strength. When the plaster sets, the whole will become one rigid stone jacket, holding the patient firmly in position. Strange to say, this petrified envelope does not genrally embarrass the breathing, though I have seen one or two exceptions to the rule. Before applying the bandages, a compress of cotton batting is applied to the lower half of the abdomen, beneath the shirt. After the plaster is hardened, the compress is removed, thus furnishing space for abdominal respiratory movements.

The apparatus can be applied in less elaborate fashion if desired. Thus, instead of suspending the patient by pulleys, three assistants can hold him horizontally face downwards by the thighs, arms and head. In this position the weight of the body causes it to settle down in the centre, thus flexing the spine backward in a desirable manner. It is not absolutely necessary to use cross-barred crinoline, as any thin, loose fabric will do. I have also seen very effective jackets applied by coating three layers of cotton flannel with wet plaster, wrapping them around the body, and confining them with an ordinary roller bandage. The patient is held meantime in a suitable position until the plaster sets.

Cases of lateral curvature can be treated by this apparatus with considerable efficiency, but its main use is for Pott's disease.

In answer to the often-repeated question, what estimate should we put upon the usefulness of the plaster jacket, I would say as follows:

Like all other useful things, it has its advantages and disadvantages. The advantages are as follows:

Cheapness. It costs next to nothing for material, though it consumes considerable time on the part of the surgeon. The cheapness enables one to apply it to numerous cases where poverty prevents the parties from paying $25 to $40 for a regular supporter.

2. Ease of construction. Any well educated physician can apply it himself however remote he may be from instrument makers. This is a very material advantage, for in order to reach an instrument maker, many patients are obliged to travel hundreds of miles, because the attempt to construct an efficient spinal apparatus from measurements sent by mail without the personal presence of the patient, is almost always a miserable failure, and the expense of the trip is often greater by far than that of the apparatus.

3. Perfection of the fitting. The plaster being applied wet, molds itself perfectly to the form, so that a "good fit" is easily obtained.

4. Efficiency. Where the deformity is situated in the lumbar or lower dorsal region, the plaster jacket has a control over it fully equal to that of any appliance ever used.

The disadvantages are as follows:

1. The jacket cannot be often changed without involving an expense for the surgeon's labor, which would soon be far greater than that of a good supporter. Hence, while wearing it, the patient is deprived of the use of his baths, somewhat to the detriment of his health. Sweat, however, does not accumulate, owing to the absorbent power of the plaster, but considerable decomposing cuticular matter accretes on the skin which cannot be removed. To a certain extent the advantages of easy removal can be obtained by foregoing those which result from carrying the plaster entirely around the body. A carapace, or "turtle shell," as Sayre calls it, may be made by putting several thicknesses of cotton flannel and plaster, strengthened by strips of perforated tin, upon the posterior half of the body and binding it on by a roller bandage. When this has hardened it can be enveloped in a jacket of cloth fitted to the body and made to lace up in front, and can be removed for bathing purposes as often as desired. The plaster portion of the apparatus not being strengthened by continuity in front, must be made pretty thick and strong, but it often serves a very good purpose. This form of the jacket has merits, but yet it has fallen, for the most part, into disuse.

2. The plaster jacket, in its original form, is only applicable to deformities existing below the level of the border of the axilla. On Pott's disease above that level it has but little effect, as the jacket cannot effectively be carried any higher. However, by the help of a mechanic, steel splints like those of a regular spinal supporter can be imbedded in the plaster and carried up high enough to apply shoulder straps, or even a head-stretch, but this is not much easier nor very much cheaper in the long run, than a regular brace.

3. The plan of suspending part of the weight of the patient by the head, involves a danger if the surgeon is at all careless. All joints of the body are liable to congenital or acquired laxity of ligaments, and the upper cervical articulations are no exception. If the surgeon chanced to have one of these relaxed cases in his suspension-sling, and by any inadvertence of himself or of his assistants, should bring too strong a tension in the head-straps, the horrible accident of a dislocation of the neck might easily occur. Great care should be taken to guard against this possibility.

From these considerations it is evident that the plaster of Paris jacket is a very valuable appliance in numerous cases, and has the especial merit of being an extempore apparatus which can be applied however remote the patient may be from instrument makers, and however impoverished he may be in purse. It has, however, some special disadvantages, and at its best, is no way superior to a well-fitted combination brace, containing the corset and the splint in one instrument. Indeed the superior convenience and tidiness of the combination brace will always cause it to be preferred by a large number of surgeons and patients.—*Edward Andrews, M.D., in Chicago Medical Journal and Examiner.*

